# Pilot study of a social network intervention for heroin users in opiate substitution treatment: study protocol for a randomized controlled trial

**DOI:** 10.1186/1745-6215-14-264

**Published:** 2013-08-19

**Authors:** Edward Day, Alex Copello, Jennifer L Seddon, Marilyn Christie, Deborah Bamber, Charlotte Powell, Sanju George, Andrew Ball, Emma Frew, Nicholas Freemantle

**Affiliations:** 1 Birmingham & Solihull Mental Health Foundation Trust, Research & Innovation Department, Radclyffe House, 66-68 Hagley Road, Birmingham B16 8PF, UK; 2 Addictions Department, National Addiction Centre, Addiction Sciences Building, 4 Windsor Walk, Denmark Hill, London SE5 8AF, UK; 3 School of Psychology, The University of Birmingham, Birmingham B15 2TT, UK; 4 Leicester City Drug & Alcohol Service, Paget House, 2 West Street, Leicester LE1 6XP, UK; 5 Health Economics, School of Health and Population Sciences, College of Medical and Dental Sciences, University of Birmingham, Edgbaston, Birmingham B15 2TT, UK; 6 Department of Primary Care and Population Health, Upper Third Floor, UCL Medical School (Royal Free Campus), Rowland Hill Street, London NW3 2PF, UK

**Keywords:** Randomized controlled trial, Social networks, Social behavior and network therapy (SBNT), Heroin use

## Abstract

**Background:**

Research indicates that 3% of people receiving opiate substitution treatment (OST) in the UK manage to achieve abstinence from all prescribed and illicit drugs within 3 years of commencing treatment, and there is concern that treatment services have become skilled at engaging people but not at helping them to enter a stage of recovery and drug abstinence. The National Treatment Agency for Substance Misuse recommends the involvement of families and wider social networks in supporting drug users’ psychological treatment, and this pilot randomized controlled trial aims to evaluate the impact of a social network-focused intervention for patients receiving OST.

**Methods and design:**

In this two-site, early phase, randomized controlled trial, a total of 120 patients receiving OST will be recruited and randomized to receive one of three treatments: 1) Brief Social Behavior and Network Therapy (B-SBNT), 2) Personal Goal Setting (PGS) or 3) treatment as usual. Randomization will take place following baseline assessment. Participants allocated to receive B-SBNT or PGS will continue to receive the same treatment that is routinely provided by drug treatment services, plus four additional sessions of either intervention. Outcomes will be assessed at baseline, 3 and 12 months. The primary outcome will be assessment of illicit heroin use, measured by both urinary analysis and self-report. Secondary outcomes involve assessment of dependence, psychological symptoms, social satisfaction, motivation to change, quality of life and therapeutic engagement. Family members (n = 120) of patients involved in the trial will also be assessed to measure the level of symptoms, coping and the impact of the addiction problem on the family member at baseline, 3 and 12 months.

**Discussion:**

This study will provide experimental data regarding the feasibility and efficacy of implementing a social network intervention within routine drug treatment services in the UK National Health Service. The study will explore the impact of the intervention on both patients receiving drug treatment and their family members.

**Trial registration:**

Trial Registration Number: ISRCTN22608399

ISRCTN22608399 registration: 27/04/2012

Date of first randomisation: 14/08/2012

## Background

There are estimated to be 306,100 users of heroin or crack cocaine in the UK [1]. There has been a large increase in investment in the management of drug dependence in the past 10 years, with the number of people in contact with treatment services in England doubling between 1998 and 2005 [2]. A key driver for this increase in provision has been a desire to reduce criminal activity, but there is now concern that treatment services have become skilled at engaging people but not at helping them to change their problematic behaviors and enter a stage of ‘recovery’ [3]. For example, research in Scotland found that 3% of people in opiate substitution treatment (OST) manage to achieve abstinence from all prescribed and illicit drugs within 3 years of commencing treatment [4]. In particular, there is concern that effective psychosocial interventions are not delivered to this population and previous attempts to develop the UK evidence base have not been successful [5].

Opioid-dependent patients receiving methadone often spend much of their time in social environments that support and directly reinforce drug use and behaviors that convey considerable risk of harm to self and others [6-9]. Patients are routinely advised to abandon their drug-using supports without having meaningful alternative social networks in place, and this typically results in the individual remaining entrenched in existing social networks. One goal of treatment interventions may be to help patients transform social networks that support drug use into those that offer competitive reinforcement for abstinence. The overall merits of this goal are illustrated by a series of studies showing that positive social supports are associated with a reduced risk of relapse to heroin and other drug use and with an overall better treatment response [10-14].

Recent UK policy developments in the drug treatment field have emphasized the role of families and communities in recovery from drug use [15,16]. Strategic documents have pointed to the low numbers of patients exiting opioid substitution programs drug-free, and the need to move beyond harm reduction and stabilization [17]. The National Treatment Agency for Substance Misuse (NTA) is ‘keen to unlock the potential for families and significant others to play an important supportive role in the recovery of individuals through their greater involvement in treatment where this is appropriate’ (NTA, 2010, page 15) [18]. Despite the available evidence, current models of drug treatment remain individually focused. It is therefore important to evaluate treatment interventions that may influence the social environment of drug users in a way that promotes a positive change in addictive behavior. The intervention needs to be feasible in routine United Kingdom National Health Service (NHS) practice and accepted by both service users and members of the clinical staff team. It also needs to impact on patients’ social networks in the way that research suggests can aid positive change in drug use and the pursuit of recovery (that is, abstinence from drugs, mental and physical health, and citizenship [19]).

Social Behavior and Network Therapy (SBNT) is an intervention developed in the UK. It integrates effective strategies from other treatment approaches and is built upon the premise that social network support for change is central to the resolution of addictive behavior [20]. The intervention facilitates the involvement of close friends and family as part of the treatment process to promote substance use change. The approach was initially targeted at alcohol problems, and was shown to be as effective and cost-effective as Motivational Enhancement Therapy in the large UK Alcohol Treatment Trial (UKATT) [21,22]. SBNT has since been adapted for use with primary heroin users. Twenty therapists from community drug services in Birmingham were trained to deliver the intervention, supported by a treatment manual and a 2-day workshop followed by video supervision. Twelve therapists delivered SBNT to 24 patients, and 3-month outcomes were measured using both quantitative and qualitative methods. The results suggested that it was feasible to train therapists to deliver SBNT, with the participating patients reporting a reduction in drug use and improved family and social relationships [23].

SBNT was initially developed to be delivered over eight sessions, although evaluation showed that the actual number of sessions attended by participants was fewer than eight, with 64% of the UKATT sample (n = 320) receiving no more than four sessions [24]. Some of the most important components of the intervention occur during the early part of the treatment: for example, drawing a social network map; contacting and inviting people; reviewing communication and interactions with significant social network members. Therefore, the intervention will be adapted for the current study to follow a four-session format, and the treatment manual used in previous pilot work will be adapted accordingly [23]. SBNT is therefore hereafter referred to as Brief Social Behavior and Network Therapy, or B-SBNT.

An important additional area that the present study aims to explore is the feasibility of recruiting family members of the patients with drug problems and assessing the impact of the drug problem on the family over time. It has been shown that family members living or in close contact with someone with an addiction problem suffer high levels of stress symptoms and are regular visitors to the primary care healthcare system [25]. To date, there has been no measurement of the potential benefits to family members of interventions focused on social networks that may include family members. Most outcome measurement has focused only on the drug-related behavior of the user. To test the feasibility of recruiting a sample of family members to assess changes in symptoms of stress over time in a confirmatory trial (either associated with the social intervention or not), we aim to recruit one family member per patient entered into the trial at baseline and to follow the sample up at 3 and 12-months post-intervention.

The overall aim of this early phase trial therefore is to implement, observe and assess the efficacy of a social-network intervention (B-SBNT) for both OST patients and their social network members. The trial aims to evaluate the feasibility of training NHS clinicians to deliver B-SBNT, and to assess the feasibility of recruiting and retaining patients engaged in drug treatment services to the trial. Another aim is to evaluate the feasibility of measuring changes in the health and functioning of family members before and after a psychosocial intervention.

This early phase trial will test the hypothesis that B-SBNT is more effective than a case management intervention of similar intensity or treatment as usual in reducing illicit heroin use 3 and 12 months after treatment in patients receiving OST.

In addition, an attempt will be made to recruit one family member for each participant in the trial. An important aim of this part of the study is to establish the feasibility of recruitment of family members for a future confirmatory trial. The family members recruited may or may not be involved in the B-SBNT intervention at a later stage, but will all be invited to undergo an assessment of levels of stress and associated coping behaviors with the intention of testing whether those participants receiving the network intervention show greater reductions in symptoms.

Finally, qualitative interviews will be conducted with those receiving as well as those delivering B-SBNT. For patients the focus will be on the level of satisfaction with the treatment, the perceived process of change and the helpful aspects of the therapeutic process. For therapists the focus will be on the experience of delivering B-SBNT.

## Method

### Study design

**Figure 1:**
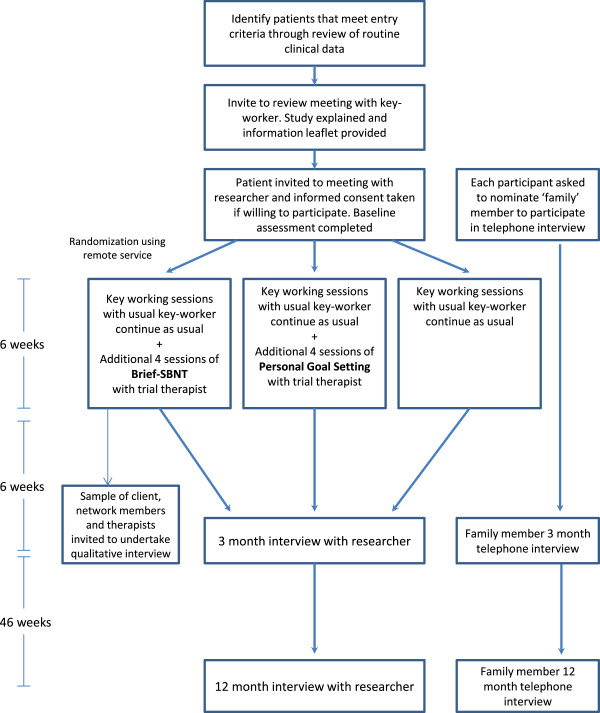
Flow diagram of the study design.

This study is an early phase, two site randomized controlled trial comparing the impact of B-SBNT, Personal Goal Setting (PGS), and treatment as usual (TAU) for patients receiving OST. The trial will be conducted within two community drug treatment teams in two UK regions: Solihull in the West Midlands and Leicester in the East Midlands. Figure 1 shows a flow diagram for the trial, consistent with the Consolidated Standards of Reporting Trials 2010 statement [26].

### Intervention

Participants will be randomized to receive one of three treatment interventions: B-SBNT plus TAU, PGS plus TAU, or a TAU control condition. Therefore, participants allocated to either of the first two groups will receive an active intervention as an adjunct to usual care provided in the service, delivered by a different therapist to the participant’s usual key-worker.

#### Brief Social Behavior and Network Therapy

Participants randomized to receive B-SBNT will be offered four 50-minute B-SBNT sessions over a maximum of 6 weeks. The treatment manual will combine the most effective components of the SBNT intervention from earlier studies [24] with elements of node-link mapping to facilitate the training and delivery of the intervention. The treatment will involve working with the patient to draw a ‘network diagram’ during the first session to identify potential social support for change that could be drawn upon during the remaining sessions. Following this, potential supportive network members identified by the participant will be approached and invited to take part in treatment sessions to enhance the social support for change in drug use. The therapist will use elements of communication skill development, coping behaviors and the development of joint activities to support the process, with the ultimate aim of building a network-supported relapse management plan.

#### Personal Goal Setting

Participants randomized to receive PGS will be offered four 50- minute sessions over a period of 6 weeks. In common with the B-SBNT intervention, therapy will be delivered according to a purpose designed manual [27]. The PGS condition is designed to control for the intensity of the treatment as well as the process and experience of receiving an intervention from a different therapist to the one delivering the routine care. This intervention will be based on the principles of node-link mapping [28] and will include a review of the participant’s current situation and future aspirations, the development of SMART (Specific, Measurable, Agreed-upon, Realistic, Time-limited) goals, and monitoring and feedback on progress in achieving these goals.

#### Treatment as usual

Participants randomized to receive TAU will continue to receive usual care with no additional therapy sessions. The participating treatment services do not specify a particular therapeutic style for working with patients, and clinicians do not follow a treatment manual. They are encouraged to use a variety of techniques, and some will have been exposed to the goal setting and SBNT techniques as part of their continuing professional development. Our research group conducted one of the few published studies describing routine treatment in OST services in Birmingham [29]. Meetings with patients occurred between weekly and fortnightly, and lasted an average of 45 minutes. Session activities fell into four broad categories, each delivered in similar amounts: case management, signposting of other services, structured psychosocial interventions, and other activities (for example, medication issues). We therefore anticipate that TAU will consist of interventions that are both less structured and less frequent than the two active treatments under test.

Prior to the commencement of the trial the elements that comprise ‘standard treatment’ in drug services at both project sites will be evaluated based on the methodology of previous research [29]. Ten clinical cases will be selected at random from each site. Electronic data will be screened for the preceding 6-week period to determine the quantity and frequency of drug treatment sessions. Short unstructured interviews will then be conducted with each key-worker to identify the content of each treatment session.

### Treatment monitoring and fidelity

To ensure that B-SBNT and PGS are delivered with sufficient fidelity and integrity, all trial therapists will be required to participate in monthly supervision meetings with members of the research team. Each therapeutic session with the therapist will be audio recorded, and a number of these recordings will be randomly selected for assessment of the fidelity of the intervention delivery by members of the research team using the UKATT Process Rating Scale [30]. This scale was developed as part of the UKATT and provides a checklist to ensure that key components of the intervention are being delivered.

The project steering group will be convened at 3-month intervals, involving the principal investigators, researchers and service user representatives. An independent expert in the field of addiction will review the data at 6-monthly intervals.

### Staff recruitment and training

This study aims to test the feasibility of training NHS staff to deliver a social network intervention. Therefore, clinical staff will be asked to volunteer to participate in the study with no attempt made to randomly select therapists. A minimum of two clinicians per treatment condition is required at each project site, but more staff will be trained if available. Training will follow the format adopted in previous pilot work in this area [23]. Each active intervention will be delivered according to a purpose-designed treatment manual. The treatment manual for B-SBNT will be based on a manual from previous research [23] adapted for a four-session format. The PGS intervention will follow the manual developed as part of the National Treatment Agency *Routes to Recovery* initiative [27]. There will be an initial 1 day training session to introduce staff to the key concepts and procedures involved in each treatment intervention, and all staff in the B-SBNT condition will be required to pilot the methods with one clinical case prior to the commencement of the trial. The trial will only commence once it has been established that B-SBNT is being delivered with sufficient fidelity, and supervision will be provided for both active treatment conditions on a monthly basis.

### Participant recruitment and randomization

Four entry criteria must be met for inclusion in the study: the patient must be 18 years or older; the patient must have been receiving OST (with either methadone or buprenorphine) continuously for a period of at least 12 months; the patient must report use of heroin on at least 1 day in the preceding 28 days; and the patient must give informed consent to participation.

There will be three exclusion criteria: the patient has refused to allow their anonymized data to be sent to the National Drug Treatment Monitoring Service, thus preventing initial screening (see below); the patient refuses to give informed consent to participation in the study; and the patient has a severe co-morbid mental or physical health issue that prevents them participating in treatment sessions.

All patients receiving treatment from a specialist substance misuse treatment service in England are asked to complete the Treatment Outcome Profile every 3 to 6 months [31], which includes questions about use of illicit opiates. These data are stored as part of the patient’s electronic record along with details of length of time in treatment, unless the patient has refused consent to share this information. These data will be interrogated by the research team every 6 weeks to identify potential participants that meet the study inclusion criteria. A list of patients meeting the criteria will be given to clinical staff in each participating team, and clinicians asked to approach the patients about the study at their next routine clinical appointment. The patient’s normal key worker will therefore provide an initial overview of the project and written information about the study. If the patient expresses an interest in taking part they will then be contacted by a member of the research team who will explain the study in more detail and invite them to sign a written consent form. Patients will be randomized and allocated to treatment intervention following completion of the baseline interview. A dynamic randomization algorithm will be used, minimizing differences in the numbers allocated to each experimental group [32]. As this is an open trial, randomization will not be stratified by investigational site as this would not be properly concealed, and will be done using a secure, remote randomization service independent of the research team.

### Assessments

Assessment will take place at baseline (prior to treatment randomization), and at 3 and 12 months following baseline assessment. Socio-demographic information (age, sex, ethnicity, employment status and living situation) will be collected at baseline only, but all other measures will be administered at all 3 time points.

#### Illicit drug use

An instant result urine toxicology test screening for metabolites of opiates will provide an objective measure of illicit drug use. This will be supplemented by an interview using sections of the Maudsley Addiction Profile (MAP) [33]. Section B records the number of days that the participant has used heroin, cocaine, benzodiazepines and alcohol in the past 28 days, and the average amount of use of each drug on a using day. Section C captures the number of days that the participant has injected drugs in the past 28 days [33]. Section E records the number of days of paid work and the amount of acquisitive crime committed in past 28 days [33]. In addition, the Leeds Dependence Questionnaire [34] will serve as a diagnostic measure of the severity of dependence and as a treatment outcome measure that works with abstinent patients. It consists of 10 questions that are summed to compute a maximum score of 30, with a higher score denoting more severe dependence.

#### Psychological and Social Functioning

The Clinical Outcome in Routine Evaluation scale [35] is a 34-item self-complete questionnaire that gives a measure of psychological morbidity across four domains: (a) well-being (4 items); (b) symptoms (12 items - depression × 4, anxiety × 4, trauma × 2, physical × 2); (c) functioning (12 items - general × 4, social × 4, and close × 4); and risk (6 items - to self × 4 or to others × 2). The Social Satisfaction Questionnaire [36] measures change in social problems. It is an eight-item self-complete questionnaire with a maximum score of 24, where higher scores represent greater satisfaction with housing, finances, and relationships.

#### Motivation to Change Behaviour

The Readiness to Change Questionnaire - Treatment Version [37] is a 15-item questionnaire, based on Prochaska and DiClemente’s stages of change model [38], that assigns drug users to Precontemplation, Contemplation, and Action stages.

#### Social Network Composition and Support

The Important People Drug and Alcohol Interview [14] is a researcher-administered adaption of the Important People and Activities measure that incorporates questions about substance abuse. Respondents are asked to provide the first name and relationship of up to 10 members of their social network who have been important to them in the last 3 months. For each network member identified, the respondent rates frequency of contact, how important the person is to them, the extent to which the person was generally supportive of them, substance use status, frequency of substance use, how this person has reacted to their substance use, and how this person has felt about them coming for treatment. The Interpersonal Support Evaluation List [39] consists of a list of 40 statements concerning the perceived availability of potential social resources. Items each fall into four 10-item subscales: tangible support, appraisal support, self-esteem support, belonging support. Each is scored between 0 and 30, with a higher score indicating greater support.

#### Therapeutic engagement

The Engagement section of the Client Evaluation of Self and Treatment [40] is a 36-item self-completion questionnaire. Each item is scored between 1 and 5, yielding four sub-scale scores representing treatment participation, treatment satisfaction, counseling rapport, and peer support.

#### Family member assessment

Each participant recruited into the trial will be asked to nominate a family member to be approached to take part in a short telephone interview at baseline, 3 and 12 months. Symptoms of stress experienced by the family member, coping behaviors used and the impact of the addiction problem on the family member will be assessed using a validated set of standardized questionnaire measures [41]. The Family Member Impact scale is a 16-item questionnaire designed to assess the extent of harmful impact that a family member perceives the relative’s drinking or drug-taking to have on them or the whole family over the previous 3-month period. Response options for each individual item (‘not at all’, ‘once or twice’, ‘sometimes’ and ‘often’) are scored 0, 1, 2 and 3, respectively. The Symptom Rating Test measures physical and psychological ill health in the general population. It is brief and easy to complete. Respondents are asked to indicate whether they have experienced each of 30 symptoms in the past 3 months using response options ‘never’, ‘sometimes’ or ‘often’ (scored 0, 1 and 2, respectively). The Coping Questionnaire measures 30 coping actions over the previous 3-month period. Respondents are given four response options for each item: ‘no’, ‘once or twice’, ‘sometimes’ and ‘often’, scored 0, 1, 2 and 3, respectively. Previous research suggests the presence of three factors (tolerant, engaged and withdrawal coping). Each factor is scored separately as a sub-scale and a total score is calculated by adding the three factor scores.

#### Qualitative assessment

A small number of participants randomized to receive B-SBNT (n = 10), family members of patients receiving B-SBNT (n = 10) and therapists trained in B-SBNT (n = 6) will be asked to participate in a short semi-structured qualitative interview with the aim of establishing the level of satisfaction with the treatment, the perceived process of change and the helpful aspects of the therapeutic process. In the case of therapists, the interviews will also assess how B-SBNT differs from standard treatment.

#### Economic evaluation

The economic evaluation will be conducted from a societal perspective, incorporating the widest measurement of costs and outcomes as possible. The base-case analysis will estimate the incremental cost-effectiveness of B-SBNT versus the two other treatment conditions using the primary outcome of abstinence of heroin. Further analyses will incorporate additional outcomes measured from the perspective of the patients and network members and will include assessment of ‘capability wellbeing’ as measured by the Investigating Choice Experiments Capability measure for Adults (ICECAP-A) [42] and health status as measured by the EQ-5D [43] at baseline, 3 months and 12 months. Health service and criminal justice service resource use will be measured using the Client Service Receipt Inventory [44], and will include number of general practitioner visits, outpatient and inpatient visits, community care and drug-related services, criminal justice service costs and medication use. Unit costs will be obtained from standard sources.

### Sample size

As this is an early phase study, a formal sample size calculation is not appropriate. A total of 120 participants will be recruited to the trial (n = 40 per treatment condition). If the proportion of patients that stop taking heroin in the B-SBNT group is 0.3 we can then produce an approximate 95% confidence interval of 0.18 to 0.44 for this estimate; if the proportion is found to be 0.1, an approximate 95% confidence interval will be 0.01 to 0.19, showing reasonable precision.

### Data analysis

#### Trial outcome analysis

Data will be analyzed according to the intention-to-treat principle; all randomized participants will be included in the analysis irrespective of whether or not they stayed in the trial, with missing data treated as failing to achieve reduction. The primary outcome measure will be the number of days in the preceding 4 weeks that the participant has used heroin. The primary analyses will compare B-SBNT with PGS and TAU, and subjects will be analyzed using a generalized mixed model, including experimental group as an explanatory classification variable. The therapists will be included as random effects [45]. In supportive analyses we will examine sequential measurements of heroin use over 28-day periods as repeated measures, using appropriate error structures, including a measure of patient adherence to randomized therapy over time. Analysis of continuous secondary outcomes will be conducted using analogous statistical models, including changes in level of drug-related problems (such as injecting drug use, criminal activity, and psychological symptoms), severity of drug dependence, motivation for drug abstinence, level of social satisfaction, and level of therapeutic engagement. A further important research outcome will be changes in social network structure and function, and level of general social support. The major analyses will be pre-specified in a statistical analysis plan completed prior to database lock. Analyses will be conducted in SAS 9.2 or above (SAS Institute Inc., Cary, NC, USA; http://www.sas.com/software/sas9; accessed 11/07/13)

#### Qualitative analysis

In line with our previous work involving qualitative evaluation of SBNT [46], interviews will be recorded and transcribed verbatim for analysis. Thematic analysis will be used to analyze the data, and the findings will be presented to a selection of the original participants to check the validity of the resulting interpretation [47].

#### Economic analysis

The economic analyses will focus on logistical issues such as the acceptability, feasibility and reliability of the data collection instruments in this trial population. Information will be collected on length of time taken to complete each instrument, analysis of missing responses and exploration of psychometric properties. Descriptive statistics will be computed for the EQ-5D and the ICECAP-A, and the within-individual difference in mean quality-of-life scores will be tested. The evaluation will be conducted from both a healthcare and a societal perspective. The analyses will be a within-trial cost-effectiveness analysis based on the primary outcome of ‘abstinence from heroin’ (for example, the difference in primary outcome between the intervention arm and the other two arms of the trial). A secondary analysis will be a cost-utility analysis using quality-adjusted life years as the outcome. A decision-tree model [48] will be used that will adopt an incremental approach and focus on the differences in cost and outcomes between the trial arms. Appropriate one-way and multi-way deterministic sensitivity analysis will be carried out to test the robustness of the results [49]. The choice of variables to assess as part of the sensitivity analysis will be confirmed when the data collection is complete but will focus on the modeling variables which are the most uncertain and for which there is the greatest amount of sampling variability.

### Ethics

This study has received approval from the National Research Ethics Committee: The Black Country (REC number: 12/WM/0046; approved 08/05/2012).

## Discussion

The National Institute for Health and Clinical Excellence has highlighted the evidence for psychosocial treatments for heroin dependence [50], and family/social network interventions are promising for two reasons: (1) by involving family and friends in the treatment process there are opportunities for generalization of the effects beyond an episode of professional treatment; and (2) treatment may reduce the considerable physical and psychological health burden of drug misuse on family and friends.

The B-SBNT intervention has been specifically adapted to make it more suitable for use within UK drug treatment services by using the elements found to be most important in the process of drug-use change [24]. This study aims to establish the feasibility of delivering a social network-based treatment for patients receiving OST within NHS drug services, as well as the impact of the intervention on both short (3 months) and longer-term outcome (12 months) for patients and their family members. Evaluation of the efficacy of B-SBNT will involve quantitative and qualitative methodology, and the trial will involve a comprehensive economic evaluation.

This study will recruit and follow-up family members of patients in UK NHS drug treatment within a randomized controlled trial design, and the results will inform the feasibility of recruitment of family members in a larger confirmatory trial with the potential to help further our understanding of the impact of drug use on family members, as well as the benefits to family members from inclusion in the drug treatment process.

The study will quantify various elements of uncertainty around the delivery of social network interventions in the OST population, and the measurement of its effectiveness: the feasibility of recruiting and training staff (that is, number willing to be trained, levels of attendance at training and supervision sessions, degree of adherence to the treatment manuals, level of staff satisfaction with the training and the interventions); the feasibility of recruiting patients (that is, number wishing to participate, attendance at assessment sessions, attendance at intervention appointments, client satisfaction with B-SBNT); the feasibility of recruiting network members into the treatment process (that is, numbers agreeing to participate, attendance at appointments, level of acceptance of B-SBNT treatment); the feasibility of recruiting family members to evaluate the impact of the intervention on their health (that is, number of family members recruited and interviewed, acceptance of the measures of symptoms and coping); and the feasibility of employing the chosen research procedures in clinical services (that is, recruitment methods, including giving information and obtaining consent), randomization, intervention delivery and outcome measurement).

In order for this study to lead to a definitive trial it will need to provide an indication of positive change in the main outcome measure for the social network intervention. However, it will also be important to know the standard deviation of this measure to determine if the required sample size for a definitive trial is realistic. The study will tell us whether participating centers are likely to recruit a sufficient number of participants to deliver a full trial within a reasonable timescale.

Research demonstrates significantly better outcomes for drug-using individuals, such as reduced risk of drug relapse and better response to treatment, when there is positive social support for change [10-14]. However, at present no available model exists to implement a family and social intervention in routine practice in the UK NHS drug treatment system. Furthermore, there is very limited knowledge of the composition and potential availability of supportive social networks to people in drug treatment services. This study is an important step in the development of evidence and aims to address these gaps in the literature. It is hoped that the results of the study will help to inform future service provision in drug treatment services for patients receiving OST.

## Trial status

The trial is currently in the recruitment phase.

## Abbreviations

B-SBNT: Brief Social Behavior and Network Therapy; EQ-5D: European Quality of Life; : ICECAP-A,; MAP: Maudsley Addiction Profile; NHS: National Health Service; NTA: National Treatment Agency; OST: opiate substitution therapy; PGS: Personal Goal Setting; SBNT: Social Behavior and Network Therapy; TAU: treatment as usual; UKATT: UK Alcohol Treatment Trial.

## Competing interests

The authors declare that they have no competing interests.

## Authors’ contributions

ED is the chief investigator for the project. ED, AC and JLS drafted the manuscript. ED, AC, MC, SG, AB, EF and NF contributed to the design of the study. JLS coordinated the implementation of the project. JLS, DB and CP were involved in the collection of data. All authors read and approved the final manuscript.
